# Higher Income and Integration into the Workforce Are the Main Factors Associated with Quality of Life in Acromegalic Patients in Northeastern Brazil

**DOI:** 10.1155/2018/6135080

**Published:** 2018-02-27

**Authors:** Adriana Maria GuimarãesSá, Pedro Antônio Muniz Ferreira, Marinilde Teles Souza, Gilvan Cortês Nascimento, Sabrina da Silva Pereira Damianse, Viviane Chaves de Carvalho Rocha, Manuel dos Santos Faria, Adalgisa de Souza Paiva Ferreira

**Affiliations:** ^1^Graduate Program in Public Health, Department of Public Health, The Federal University of Maranhão (UFMA), São Luís, MA, Brazil; ^2^Presidente Dutra University Hospital of The Federal University of Maranhão (HUUPD-UFMA), São Luís, MA, Brazil; ^3^Clinical Research Center, UFMA, São Luís, MA, Brazil; ^4^Graduate Program in Child and Adult Health, Department of Medicine, UFMA, São Luís, MA, Brazil; ^5^Graduate Program in Public Health, Department of Public Health, UFMA, São Luís, MA, Brazil

## Abstract

**Objective:**

To identify the factors associated with quality of life in patients with acromegaly with follow-up at the referral service in neuroendocrinology of the state of Maranhão, northeast Brazil.

**Methods:**

The Acromegaly Quality of Life Questionnaire (Acro-QoL) was used. Factors independently associated with quality of life were identified using multivariate linear regression, with *p* values < 0.05 considered significant.

**Results:**

The multivariate linear regression analysis indicated a positive association between being integrated into the job market and quality of life scores in the overall domain (*β* = 0.288, *p* = 0.003), psychological domain (*β* = 0.291, *p* = 0.032), and personal relationship domain (*β* = 0.314, *p* = 0.019). We also observed a positive association with income and the quality of life scores in all domains as follows: overall domain (*β* = 0.037, *p* = 0.003), physical domain (*β* = 0.988, *p* = 0.001), psychological domain (*β* = 0.342, *p* = 0.008), physical appearance domain (*β* = 0.270, *p* = 0.049), and personal relationship domain (*β* = 0.315, *p* = 0.012).

**Conclusion:**

For patients with acromegaly living in one of the least developed regions of Brazil, integration into the job market and a higher income were associated with a better quality of life.

## 1. Introduction

Patients with acromegaly produce high levels of growth hormone (GH) and insulin-like growth factor 1 (IGF-I), which cause facial changes, a coarse physical appearance, and enlargement of the internal organs. These effects may gradually lead to cardiovascular disease, psychological disorders, and osteoarticular damage, which limit the physical skills necessary for the maintenance of the activities of daily living (ADLs) and consequently compromise quality of life (QoL) [1, 2].

In these individuals, factors such as being female, duration of the disease [3], age, and radiotherapy [4] have been negatively associated with QoL. Although improvement in QoL may be achieved with drug therapy or surgery [5, 6], physical and psychological morbidities may impair QoL, even in acromegalic patients in long-term biochemical remission (i.e., patients with controlled disease) [7, 8]. Therefore, the data on the correlation between disease control and QoL in acromegaly are controversial [9–14].

Despite the growing interest in the QoL of patients with acromegaly, no studies have evaluated this parameter in the Brazilian population. The availability of these data in a developing country such as Brazil, especially in disadvantaged regions of Brazil such as the northeast, is essential because socioeconomic conditions may interfere more significantly with the QoL of these individuals than of individuals living in developed countries. Few studies indexed in MEDLINE were found on this subject in Latin America. We found a single case report from Argentina [5] and one study with 50 patients from Mexico [8]. In this study, we aimed to identify the factors associated with quality of life in patients with acromegaly with follow-up at the referral service in neuroendocrinology of the state of Maranhão, northeast of Brazil.

## 2. Materials and Methods

### 2.1. Subjects and Study Design

This cross-sectional study was conducted at the only public reference service in neuroendocrinology in the state of Maranhão at the Presidente Dutra University Hospital (Hospital Universitário Unidade Presidente Dutra (HUUPD)) and the Clinical Research Center (Centro de PesquisaClínica (CEPEC)) of the same hospital. This is part of a doctorate thesis carried out in the Post-graduation Program in Public Health at the Federal University of Maranhão [15]. The data were collected between April 2015 and July 2015. All 75 patients with a diagnosis of acromegaly and follow-up at the hospital in April 2015 were evaluated. The routine of the service was to evaluate patients every three months with recording of clinical and laboratory data (IGF-I and GH levels). The inclusion criteria were individuals older than 18 years who were in clinical follow-up for at least six months. The exclusion criteria were the presence of active neoplasia, severe cardiovascular disease (unstable coronary artery disease or heart failure type NYHA III-IV), pregnancy or breastfeeding, and severe depression.

Under the requirements of Resolution 466/2012 of the Brazilian National Health Council, the project was approved by the Research Ethics Committee under opinion number 1.027.553. Informed consent was obtained from all individual participants included in the study.

The data collection instruments used were a questionnaire containing socioeconomic variables [sex (male and female), age (in years), marital status (with or without a partner), education level (in years), monthly family income (number of minimum wages), and occupation (integrated or not into the workforce, i.e., economically active or not)], diagnosis [time since diagnosis (in months), presence of comorbidities, and tumor size (macro or micro)], treatment [use of specific medications, type of medications used, performance of surgery, number of surgeries performed, time since surgery (in months), and performance of radiotherapy)], and disease control. Additionally, we applied the Acromegaly Quality of Life Questionnaire (Acro-QoL), which contains 22 questions divided into two items [physical characteristics (8 items) and psychological characteristics (14 items) ranging from 0 to 110]; this questionnaire evaluates the QoL of patients with acromegaly [16].

In order to determine the monthly family income, a self-declaration was collected from the patient, adding the income of all individuals living in the same household and later categorizing them into minimum wage numbers. According to the Ministry of Labor and Employment, the last adjustment of minimum wage in the year 2014 was the amount of R$ 724.00 reais (approximately 290 US dollars).

### 2.2. Laboratory Assays

Serum IGF-I levels were analyzed using a solid-phase enzyme labeled chemiluminescent immunometric assay with an IMMULITE 1000 analyzer (Siemens Healthcare Diagnostics Products Ltd., Llanberis, Gwynedd, UK) with an intra-assay coefficient of variation (CV) of 3.1%–4.3%. The IGF-I international reference preparation was 87/518. GH serum levels were determined using a solid-phase two-site immunometric chemiluminescent assay with an IMMULITE 1000 analyzer (Siemens Healthcare Diagnostics Products Ltd., Llanberis, Gwynedd, UK) with inter- and intra-assay CVs of 5.5%–6.2% and 5.3%–6.5%, respectively. The international reference preparation for GH was 98/574.

The IGF-I and GH levels obtained in the last measurement were considered for the definition of disease control. The controlled disease cases were defined as those in which the patient presented a baseline GH level < 1 mg/L and an IGF-I level within the normal range for their age and sex [17].

### 2.3. Statistical Analysis

The descriptive analysis and statistical tests were performed using Statistical Package for the Social Sciences (SPSS) software version 23.0. The *p* values < 0.05 were considered significant. The numerical variables are presented as means, standard deviations, and maximum and minimum values, and the categorical variables are presented as frequencies and percentages. Cronbach's alpha coefficient was determined to assess the internal consistency of the Acro-QoL data. A reasonable level was considered when the alpha was ≥0.7 [18].

A comparative analysis was conducted between the mean Acro-QoL scores for sex, marital status, occupation, comorbidities, tumor size, performance of surgery, use of specific medications, and disease control. Correlations were also established between the Acro-QoL scores and age, years of education, number of minimum wages, time since diagnosis, length of use of medications, time since surgery, and number of surgeries.

Numerical variables were compared using Student's *t*-test, and correlations were identified using Pearson's test. The factors independently associated with QoL were identified by multivariate linear regression using the variables with *p* values < 0.05 in at least one domain as the independent variables in the bivariate analysis. The independent variables were the number of minimum wages (monthly family income), occupation (economically active or not), and education level (years of schooling), and the dependent variables were the isolated scores of the different Acro-QoL domains adjusted for sex and age.

## 3. Results

The state reference service in neuroendocrinology of the HUUPD contained the records of 75 patients with acromegaly, of whom 64 were in follow-up. After applying the inclusion and noninclusion criteria, the 57 participants who were eligible for the study were evaluated.

Thirty-six (63.1%) patients were women. The mean age was 53.52 ± 12.20 years, 28 (49.1%) patients were married or in a stable relationship, and the mean schooling was 9.38 ± 4.33 years. Only 25 (43.8%) patients were economically active. The mean monthly income was 3.51 ± 3.90 minimum wages, the mean time since diagnosis was 90.64 ± 58.69 months, 12 (21.8%) patients were hypertensive and had diabetes, and 41 patients (72%) had pituitary macroadenoma. Most (43.0–75.4%) of the patients were using octreotide LAR, 18 (31.5%) were using cabergoline, 34 (59.6%) had undergone surgery, 33 (99%) had undergone surgery via the transsphenoidal route, 27 (79.4%) were subjected to one surgery, and 3 (5.2%) had undergone radiotherapy (Table 1). The mean lengths of use of octreotide LAR and cabergoline were 84.22 ± 48.84 and 86.91 ± 29.35 months, respectively, and the mean times since surgery and radiotherapy were 73.12 ± 37.70 and 98.21 ± 10.44 months, respectively.

The means and standard deviations of the Acro-QoL domains and the corresponding Cronbach's alpha values are shown in Table 2. Almost all of the domains showed alpha values greater than 0.70, which indicated good internal consistency.

The comparison of the mean scores of the Acro-QoL domains between the sexes indicated that marital status, presence of comorbidities, tumor size, performance of surgery, and disease control were not significantly different. The mean Acro-QoL scores were higher for the overall domain (*p* = 0.012), psychological domain (*p* = 0.009), and personal relationship domain (*p* = 0.003) among the patients who were economically active (integrated into the workforce) and higher in the physical capacity domain (*p* = 0.031) among individuals who did not use any specific medications (Tables 3 and 4).

No correlation was observed between the Acro-QoL scores for age, time since diagnosis, length of use of specific medications, and time since surgery; however, a positive correlation was observed between years of schooling and the score on the personal relationship domain (*p* = 0.023). Additionally, a positive correlation was detected between the monthly income and the scores of all evaluated domains as follows: overall domain (*r* = 0.469, *p* = 0.001), physical domain (*r* = 0.516, *p* = 0.001), psychological domain (*r* = 0.432, *p* = 0.001), physical appearance domain (*r* = 0.335, *p* = 0.011), and personal relationship domain (*r* = 0.415, *p* = 0.001) (Tables 5 and 6).

The multivariate linear regression analysis adjusted for sex and age indicated a positive association between income and the QoL scores in all domains as follows: overall (*β* = 0.0376, *p* = 0.003), physical (*β* = 0.988, *p* = 0.001), psychological (*β* = 0.342, *p* = 0.008), physical appearance (*β* = 0.270, *p* = 0.049), and personal relationships (*β* = 0.315, *p* = 0.012). The patients who were integrated into the workforce also had better QoL scores in the overall domain (*β* = 0.288, *p* = 0.003), psychological domain (*β* = 0.291, *p* = 0.032), and personal relationship domain (*β* = 0.314, *p* = 0.019) (Table 7).

## 4. Discussion

Patients with acromegaly undergo significant physical and psychological changes that can impact their QoL. This study evaluated acromegalic patients with follow-up at the only specialized reference service in Maranhão (northeast Brazil). The study contained a representative sample of patients with this rare disease and identified a better QoL in the Acro-QoL scores among individuals integrated into the workforce (economically active), and this quality of life increased proportionally according to their monthly income. Cronbach's alpha coefficients indicated the reliability of the scores and demonstrated reasonable internal consistency of the Acro-QoL in patients with acromegaly, as reported in the studies by Webb et al. [16, 19].

The assessment of QoL using the Acro-QoL indicated that individuals who were employed had higher scores for the overall domain, psychological domain, and personal relationship domain in the bivariate analysis. This result was maintained in the multivariate regression analysis after adjustment for sex and age. Being part of the workforce is a factor that is historically associated with a better QoL [20–23]. Some studies have shown a strong relationship between a poor QoL/health and unemployment [24–26]. The study by Giatti et al. [23] indicated that unemployment was associated with increased morbidity and mortality and the adoption of behaviors that could negatively affect health.

This association was also found in patients with chronic diseases, as reported in studies conducted in other countries [27–29] wherein unemployed patients presented a worse quality of physical and mental life. Similar results were reported by Porter et al. [27], who evaluated 639 patients with hypertensive chronic kidney disease in Chicago. Morrisroe et al. [28] evaluated 1587 patients with systemic sclerosis in Australia and reached the same conclusion using the short-form 36 (SF-36) questionnaire. Zhang et al. [29] interviewed 20,700 families diagnosed with high blood pressure in China and found the same association using the Euro-QoL 5D (EQ-5D) questionnaire.

In Brazil, this finding has been confirmed in healthy subjects and subjects with morbidities [30–34]. Similarly, Flor et al. [34] investigated 12,423 participants in the southeast and north of Brazil and found that individuals who were not part of the workforce presented worse physical and mental qualities of life. However, to the best of our knowledge, no studies have evaluated the relationship between QoL and being economically active in individuals with acromegaly. Since this study identified that being in the job market has been associated with higher scores of QoL, in an independent manner, especially as far as psychological and interpersonal domains are concerned, it is feasible to infer that among acromegalic patients who are individuals that often show typical alterations in appearance, being active jobwise may bring about a sense of well-being which seems to be rather relevant to maintain better levels of QoL. Furthermore, being economically active may be justified by fewer sequelae enabling their position within the job market and, therefore, better levels of QoL.

The second factor independently associated with a better QoL in all Acro-QoL domains in this study group was monthly income. The higher the income the higher the QoL scores were. This association has been consistently reported in both healthy individuals and patients with chronic diseases [35–39]. Several studies conducted in Brazil on the QoL of patients with chronic diseases reported that patients with higher incomes had a better QoL. This association was observed in patients with hypertension (Carvalho et al. [40]), HIV (da Cunha et al. [41]), chronic liver disease (Souza et al. [42]), and chronic kidney disease in the predialysis phase (Lemos et al. [43]).

To the best of our knowledge, no studies in Brazil have evaluated the correlation between income and the QoL of patients with acromegaly. Because the Acro-QoL takes into account the physical aspects in most domains, we were surprised to find that a higher income was independently associated with a higher QoL score because physical sequelae occur regardless of the socioeconomic status of the individual. Therefore, we can speculate that individuals with higher incomes in this population have easier access to health services, which favors early diagnosis, causes fewer sequelae, and decreases the deformities caused by the disease and consequently improves the QoL scores. This factor may be important in developing countries such as Brazil, particularly in disadvantaged regions such as the northeast because these regions have lower municipal human development indices (HDI-M) [44]. A low socioeconomic status is historically related to limited access to health care and the absence of regular care [45]. This status is also present among patients with acromegaly; therefore, individuals with limited access to health services may not benefit from an early diagnosis, which worsens the morphological and physiological changes associated with the disease [46] and favors worse QoL scores.

In this study, we could not find an association between the QoL scores of the Acro-QoL and disease control. This result may be due to the irreversibility of the physical limitations (deformities, pain, and comorbidities) that affect the QoL. Because these limitations were present in the patients at diagnosis, disease control might have no influence on the outcome. Other studies have also found that the normalization of the GH and IGF-I levels, which reflect disease control, is not associated with higher QoL levels compared with healthy individuals [4] or with individuals with uncontrolled disease [47–49].

One of the limitations of this study was its cross-sectional design; therefore, the study did not take into account the variability of health-related QoL over time (i.e., before and after the control of the disease). However, other cross-sectional studies using the Acro-QoL found similar results, including the lack of association of sex, age, disease control, and radiotherapy [3, 12, 46, 48] with QoL, suggesting that these results were consistent and represented the reality of this population.

## 5. Conclusion

In conclusion our results helped clarify the socioeconomic factors associated with the QoL of patients with acromegaly in a population with a low level of human development. The finding that patients who were integrated into the workforce had improved psychological- and personal relationship-related QoL levels despite the difficulties encountered in the execution of ADLs due to the physical deformities caused by the disease will strengthen the development of measures aimed at maintaining acromegalic patients in the labor market.

Furthermore, the finding that higher income was associated with better QoL scores in all domains suggested that individuals with higher incomes in populations with limited access to public health services may have the opportunity for an earlier diagnosis, which helps prevent the marked deformities caused by the disease. Thus, social inequalities are also prevalent in individuals with rare diseases such as acromegaly.

## Figures and Tables

**Table 1:** Socioeconomic variables, diagnosis, comorbidities, and treatment of patients with acromegaly, São Luís, state of Maranhão, Brazil, 2015.

Variables	
Sex (male/female) *n* (%)	21/36 (36.8/63.2)
Age (years)^∗^	53.52 ± 12.20
Marital status *n* (%)	
Single	17 (29.8)
Married	25 (43.9)
Stable union	03 (05.3)
Widowed	06 (10.5)
Divorced	06 (10.5)
Education level (years)^∗^	9.38 ± 4.33
Occupation (%)	
Active	25 (43.9)
Retired with remunerated activity	01 (01.7)
Unemployed	05 (08.8)
Retired/pensioner	19 (33.3)
Recipient of health benefits	07 (12.3)
Monthly income (minimum wages)	3.51 ± 3.90^∗^
Time since diagnosis (months)	90.64 ± 58.69^∗^
Comorbidity *n* (%)	
Hypertension	10 (17.5)
Diabetes	04 (07.1)
Hypopituitarism	03 (05.4)
Hypertension and diabetes	12 (21.3)
Hypertension, diabetes, and hypopituitarism	17 (28.3)
None	11 (20.4)
Tumor size (micro/macro/unknown)	15/41/01 (26.3/72.0/1.7)
Use of medications (%)	
Cabergoline	05 (08.8)
Octreotide LAR	30 (52.7)
Cabergoline and octreotide LAR	13 (22.8)
None	09 (15.7)
Surgery (yes/no)	34/23 (59.65/40.35)
Surgical route (transsphenoidal/transcranial/both)	28/01/05 (82.3/2.9/14.8)
Time since surgery (months)^∗^	73.12 ± 37.70
Number of surgeries (one/two or more)	27/07 (79.4/20.6)
Radiotherapy (yes/no)	03/54 (5.3/94.7)

^∗^Values are expressed as the mean and standard deviation.

**Table 2:** Mean scores of the Acro-QoL domains from patients with acromegaly, São Luís, state of Maranhão, Brazil, 2015.

Acro-QoL domains	Mean	Standard deviation	Minimum	Maximum	Cronbach's alpha
Physical	26.82	08.57	08	40	0.73
Psychological	47.01	14.81	14	70	0.94
Appearance	22.59	07.52	08	40	0.68
Personal relationships	24.61	09.11	07	35	0.76
Total Acro-QoL score	74.14	22.0	22	110	0.91

**Table 3:** Mean comparison test for the Acro-QoL domain scores for sociodemographic variables and tumor size, São Luís, state of Maranhão, Brazil, 2015.

Acro-QoL domains (score)	Sex			Marital status			Economically active			Tumor size^∗∗^		
	Male (*n* = 21)	Female (*n* = 36)	*p* value	With a partner (*n* = 28)	Without a partner (*n* = 29)	*p* value	Yes (*n* = 26)	No (*n* = 31)	*p* value	Micro (*n* = 15)	Macro (*n* = 41)	*p* value
Total	77.38 ± 19.6	72.25 ± 23.34	0.401	77.25 ± 21.30	71.33 ± 22.59	0.314	82.62 ± 16.25	67.97 ± 23.74	0.012^∗^	77.66 ± 21.77	73.24 ± 22.35	0.512
Physical	28.19 ± 8.01	26.03 ± 8.9	0.363	27.15 ± 6.98	26.53 ± 9.91	0.790	29.33 ± 7.22	25.0 ± 9.11	0.059	29.40 ± 9.39	26.07 ± 8.22	0.202
Psychological	49.29 ± 13.54	45.69 ± 15.53	0.382	49.26 ± 14.88	45.00 ± 14.7	0.282	52.96 ± 10.76	42.70 ± 15.97	0.009^∗^	47.87 ± 14.28	46.90 ± 15.29	0.833
Appearance	23.0 ± 6.86	22.36 ± 7.97	0.760	23.41 ± 7.45	21.87 ± 7.64	0.445	24.62 ± 6.88	21.12 ± 7.85	0.083	23.20 ± 7.38	22.37 ± 7.75	0.719
Personal relationships	26.33 ± 8.72	23.61 ± 9.31	0.281	26.22 ± 8.93	23.17 ± 9.19	0.209	27.75 ± 5.91	21.61 ± 9.91	0.003^∗^	25.33 ± 8.85	24.56 ± 9.32	0.782

^∗^Significant at *p* < 0.05. ^∗∗^One datum was lost.

**Table 4:** Mean comparison test for the Acro-QoL domain scores for comorbidities, therapy, and disease control, São Luís, state of Maranhão, Brazil, 2015.

Acro-QoL domain	Comorbidities			Surgery			Use of medication			Disease		
	Yes (*n* = 46)	No (*n* = 11)	*p* value	Yes (*n* = 34)	No (*n* = 23)	*p* value	Yes (*n* = 48)	No (*n* = 9)	*p* value	Controlled (*n* = 21)	Not controlled (*n* = 36)	*p* value
Total	72.67 ± 22.01	80.27 ± 21.87	0.538	74.73 ± 22.71	73.26 ± 21.37	0.806	73.25 ± 21.9	78.88 ± 22.86	0.485	79.28 ± 20.51	71.13 ± 22.55	0.180
Physical	26.48 ± 7.49	28.27 ± 11.18	0.308	27.85 ± 9.04	25.30 ± 7.78	0.275	25.77 ± 7.91	32.44 ± 10.24	0.031^∗^	29.04 ± 8.25	25.52 ± 8.61	0.136
Psychological	45.61 ± 14.92	52.91 ± 13.36	0.144	46.94 ± 15.52	47.13 ± 14.03	0.963	46.88 ± 14.8	47.78 ± 15.74	0.869	49.47 ± 13.51	45.58 ± 15.52	0.343
Appearance	21.78 ± 7.58	26.0 ± 6.52	0.095	22.15 ± 7.92	23.26 ± 7.02	0.588	22.46 ± 7.64	23.33 ± 7.26	0.752	24.52 ± 7.98	21.47 ± 7.12	0.141
Personal relationships	24.07 ± 9.49	26.91 ± 7.3	0.357	24.79 ± 8.92	24.35 ± 9.59	0.858	24.65 ± 9.02	24.44 ± 10.17	0.952	25.42 ± 7.82	24.13 ± 9.87	0.611

^∗^Significant at *p* < 0.05.

**Table 5:** Correlation between the Acro-QoL domain scores and the socioeconomic status and disease diagnosis variables, São Luís, state of Maranhão, Brazil, 2015.

Acro-QoL domain (score)	Age (years)		Education (years)		Number of minimum wages	
	Coefficient	*p* value	Coefficient	*p* value	Coefficient	*p* value
Total	0.213	0.111	0.177	0.189	0.469	0.001^∗^
Physical	0.249	0.062	0.072	0.594	0.516	0.001^∗^
Psychological	0.155	0.248	0.220	0.100	0.432	0.001^∗^
Appearance	0.191	0.154	0.064	0.639	0.335	0.011^∗^
Personal relationships	0.111	0.410	0.302	0.023^∗^	0.415	0.001^∗^

Pearson correlation coefficient. ^∗^Significant at *p* < 0.05.

**Table 6:** Correlation between the Acro-QoL domain scores and the disease diagnosis and treatment variables, São Luís, state of Maranhão, Brazil, 2015.

Acro-QoL domain (score)	Time since diagnosis (months)		Length of use of octreotide LAR (months)		Length of use of cabergoline (months)		Time since surgery (months)		Number of surgeries	
	Coefficient	*p* value	Coefficient	*p* value	Coefficient	*p* value	Coefficient	*p* value	Coefficient	*p* value
Total	−0.132	0.328	−0.138	0.379	−0.078	0.566	0.062	0.649	−0.195	0.262
Physical	−0.134	0.320	−0.190	0.222	0.061	0.650	−0.040	0.770	−0.049	0.779
Psychological	−0.103	0.447	−0.104	0.508	−0.166	0.216	0.118	0.388	−0.251	0.145
Appearance	−0.105	0.439	−0.097	0.535	−0.115	0.393	0.110	0.421	−0.117	0.503
Personal relationships	−0.065	0.628	−0.060	0.703	−0.166	0.216	0.117	0.392	−0.333	0.050

Pearson correlation coefficient.

**Table 7:** Multivariate linear regression analysis of the factors associated with the quality of life of patients with acromegaly, São Luís, state of Maranhão, Brazil, 2015.

Acro-QoL domain (score)	Age (years)		Sex (male/female)		Education level (years)		Number of minimum wages		Economically active (yes/no)	
	*β* (95% CI)	*p* value	*β* (95% CI)	*p* value	*β* (95% CI)	*p* value	*β* (95% CI)	*p* value	*β* (95% CI)	*p* value
Total	0.196 (−0.08 to 0.79)	0.113	−0.005 (−11.5 to 11.1)	0.971	0.082 (−0.37 to 0.76)	0.498	0.376 (0.75–3.49)	0.003^∗^	0.288 (1.29–24.1)	0.030^∗^
Physical	0.195(−0.03 to 0.30)	0.113	−0.051 (−5.29 to 3.49)	0.683	−0.016 (−0.23 to 0.20)	0.897	0.445 (0.45–1.51)	0.001^∗^	0.205 (−0.90 to 7.97)	0.116
Psychological	0.152 (−0.11 to 0.48)	0.228	0.001 (−7.76 to 7.81)	0.995	0.123 (−0.19 to 0.59)	0.322	0.342 (0.35–2.24)	0.008^∗^	0.291 (0.77–16.52)	0.032^∗^
Appearance	0.167(−0.06 to 0.27)	0.221	0.038 (−3.70 to 4.870)	0.786	−0.003 (−0.21 to 0.21)	0.982	0.270 (0.003–1.04)	0.049^∗^	0.236 (−0.76 to 7.90)	0.105
Personal relationships	0.131(−0.08 to 0.28)	0.288	−0.009 (−4.8 to 4.53)	0.199	0.19 (−0.04 to 0.43)	0.106	0.315 (0.17–1.30)	0.012^∗^	0.314 (1.00–10.48)	0.019^∗^

Multivariate linear regression analysis was conducted for the variables age, sex, educational level, number of minimum wages, and employment status. These data are shown as the standardized *β* coefficient of the independent predictive factors for the subscales, confidence intervals (CIs), and *p* values. ^∗^Significant at *p* < 0.05.
